# Seeking optimization of LT4 treatment in patients with differentiated thyroid cancer

**DOI:** 10.1007/s42000-022-00376-9

**Published:** 2022-06-02

**Authors:** Ilaria Stramazzo, Silvia Capriello, Alessandro Antonelli, Poupak Fallahi, Marco Centanni, Camilla Virili

**Affiliations:** 1 Department of Medico-Surgical Sciences and Biotechnologies, ‘‘Sapienza’’ University of Rome, Latina, Italy; 2 Endocrine Unit, AUSL Latina, Latina, Italy; 3 Department of Surgical, Medical and Molecular Pathology and Critical Area, University of Pisa, Pisa, Italy; 4 Department of Translational Research and New Technologies in Medicine and Surgery, University of Pisa, Pisa, Italy

**Keywords:** Hypothyroidism, Differentiated thyroid cancer, LT4, Liquid LT4, Softgel LT4, Dissolution

## Abstract

Levothyroxine sodium (LT4) is the mainstay treatment to replace thyroid hormonal production in thyroidectomized patients, but, depending on the aggressiveness of the cancer and on the risk of recurrence, patients with differentiated thyroid cancer may also be treated in a TSH-suppressive or semi-suppressive mode. The pathophysiological rationale for this LT4 treatment stems from the role of TSH, considered to be a growth factor for follicular cells, potentially inducing initiation or progression of follicular cell-derived thyroid cancer. Therefore, accurate tailoring of treatment, taking into account both patient characteristics (age and comorbidities) and risk of persistent/recurrent disease, is highly recommended. Furthermore, adjustments to traditional LT4 treatment should be made in thyroidectomized patients due to the lack of thyroidal contribution to whole body triiodothyronine (T_3_) concentration. Since LT4 exhibits a narrow therapeutic index and the side effects of over- and under-treatment could be deleterious, particularly in this category of patients, caution is required in dose individualization, in the mode of ingestion, and in potential pharmacological and other types of interference as well. Our aim was to analyze the current knowledge concerning LT4 dose requirements in patients with thyroid cancer according to different therapeutic approaches, taking into account a number of factors causing interference with LT4 efficacy. Specific mention is also made about the use of the novel LT4 formulations.

## Introduction

LT4 sodium is the mainstay treatment to replace production of thyroid hormone in the majority of patients who undergo thyroidectomy, depending on the extent of surgery [1]. In the case of thyroid malignancy, patients may also be treated in a TSH-suppressive or semi-suppressive mode, depending on the thyroid cancer staging. The choice is based on the major Society guidelines on differentiated thyroid cancer (DTC representing 90% of thyroid tumors) [2, 3], depending on tumor size, invasion, local or distant metastasis, and response to therapy (see below). The pathophysiological rationale for LT4 treatment stems from the role of TSH, considered to be a growth factor for follicular cells, potentially inducing initiation or progression of follicular cell-derived thyroid cancer [4]. Therefore, the goal of treatment, in particular in high-risk DTC patients, is to suppress TSH levels in order to reduce the stimulation of neoplastic cell proliferation. However, TSH suppressive treatment can induce adverse effects on heart and bone (increased cardiovascular morbidity and mortality, and increased risk of osteoporosis and fractures) [5]. Moreover, Jin et al. reported that TSH suppressive therapy is associated with neurological and psychiatric disorders (short-term memory impairment, attention impairment, word selection anomia, and depression) [6]. Therefore, accurate tailoring of treatment in any setting taking into account both patient characteristics (age and comorbidities) and risk of persistent/recurrent disease is highly recommended.

## Suppressive or replacement mode of LT4 treatment

According to the ATA guidelines [2], the concentrations of serum TSH and other criteria to be attained and to be considered when using LT4 therapy following the initial treatment (surgery with or without subsequent ^131^I therapy) or according to the risk of recurrence at follow-up are as follows: (a) < 0.1 mU/l in high-risk patients or in structural incomplete response to therapy (subsequently referred to as a target of suppressive T4 treatment); (b) 0.1–0.5 mU/l in intermediate-risk patients and in low-risk patients who have detectable serum Tg levels, in biochemically incomplete response to therapy, in patients with a high risk of recurrence with an excellent or indeterminate response to therapy, and in cases of comorbidities and/or advanced age of the above-mentioned group (subsequently referred to as a target of partially suppressive T4 treatment); and (c) 0.5–2 mU/l in low-risk patients who have undetectable serum thyroglobulin (Tg) levels, in the case of excellent or indeterminate response to therapy, especially in patients at low risk for recurrence, and in the case of comorbidities and/or advanced age of the above-mentioned intermediate risk group (b) (subsequently referred to as a target of replacement T4 treatment). A recent report stated that the percentage of physicians who would recommend TSH suppressive therapy is 80.4%, 48.8%, and 29.7%, respectively, for intermediate-risk, low-risk, and very low-risk papillary thyroid cancer [7]. Although the usefulness of TSH suppressive therapy in low-risk patients is controversial [8, 9], this treatment was initially considered to be beneficial by different studies in high-risk patients and advanced DTC [8, 10, 11]. However, more recently, the role of suppressive treatment has been reconsidered even in high-risk DTC patients. Tian T et al. [12] and Klubo-Gwiezdzinska J et al. [13], in two retrospective studies, observed that in intermediate- and high-risk PTC patients, TSH levels during follow-up did not modify the rate of disease recurrence, questioning the appropriateness of suppressive therapy in these patients. The latter findings confirmed those contained in a prospective study on 433 patients with PTC who were randomized to receive LT4 at replacement or suppressive doses (target of TSH ≤ 0.01 µU/mL) [14]. Disease-free survival rates in the two treatment arms were similar, even when high-risk subjects were analyzed separately [14].

## Target TSH in relation to extent of surgery

Due to more conservative treatment recently recommended for small unilateral DTC [15, 16], the need for LT4 treatment in patients on active surveillance and in those who underwent lobectomy is questionable. According to the ATA guidelines, in low-risk patients treated with lobectomy, TSH can be maintained between 0.5 and 2 mU/L. Therefore, LT4 treatment may be redundant provided that patients maintain their serum TSH within this target range. However, Kim et al., in a retrospective study including 256 patients with DTC who underwent lobectomy, observed that after 5 years, two-thirds of the patients needed LT4 treatment [17]. Interestingly, the presence of thyroiditis and preoperative TSH levels correlated with postoperative LT4 requirement. More recently it was confirmed that about 80% of patients who underwent therapeutic lobectomy for DTC displayed serum TSH levels beyond the recommended range [18]. Conflicting results concerning patients on active surveillance were reported. Sugitani et al. for ≥ 2 years followed 322 patients with PTMC, who exhibited no significant association between mean TSH value and tumor progression [19]. In contrast, Kim et al. reported that sustained elevation of serum TSH levels was associated with progression of papillary thyroid microcarcinoma (PTMC) progression, with a cutoff value of 2.5 mUI/L in 126 affected patients [20]. Hence, due to the conflicting results, further studies incorporating longer follow-up are required to clarify these issues.

## Estimating LT4 requirements in thyroidectomized patients

An early authoritative observation by Jonklaas and colleagues showed that normal triiodothyronine (T_3_) levels were achieved using traditional treatment in 50 patients who had undergone thyroid surgery for different reasons [21]. Since then, several studies have suggested that adjustments to traditional LT4 treatment should be made in thyroidectomized patients due to the lack of thyroidal contribution to whole body T_3_ concentration. In fact, an interesting study published in 2012 in 135 thyroidectomized DTC patients stressed that TSH-suppressive doses of LT4 are required to achieve preoperative T_3_ serum levels [22]. A study including 23 goitrous patients subsequently thyroidectomized for DTC showed that an approximately 25% higher dose of T4 than the presurgical dose is needed to achieve a TSH value similar to that before thyroidectomy [23]. Several studies examined different schedules to accurately individualize LT4 treatment following thyroidectomy: while the first studies focused only on patients’ weight, the more recent took into account specific patient characteristics, such as age, sex, BMI stratification, and body surface area as well as considering possible concomitant mineral supplementation. These reports have recently been summarized in a comprehensive review article [24]. Of note, a paper published in 2014 [25] evaluated the possible impact of genetic polymorphisms in uridine 5′-glucuronosylytansferases UGT1A1 and UGT1A3 and iodothyronine-deiodinases types 1 and 2 on requirement for TSH suppression in 268 thyroidectomized DTC patients. The authors concluded that polymorphisms in DIO1 and DIO2 had no effect on T4 dose requirement and, despite UGT1A haplotypes associated with the T4 dose, their net effect accounted for only 2% of the total variability in the DTC patients with the anthropometric characteristics representing the major determinants of LT4 requirement [25].

In the literature, the LT4 dose recommended to achieve TSH suppression has been estimated at between 2 and 2.5 µg/kg weight/day [26]. It is important to note that these doses might be overestimated since they were based on analysis of studies often carried out in unselected populations prior to careful dose tailoring becoming accepted and more widely applied.

## The problems involved in optimization of LT4 dose

The most frequently used route of LT4 administration is per os, resulting in intestinal absorption of 60 to 80% of the ingested dose [27]. Since the absorption is variable and LT4 has a narrow therapeutic index, caution is required in dose individualization and in the mode of ingestion, as well as in ensuring patient compliance [24, 28]. However, a long road lies ahead until an optimal LT4 therapeutic target is achieved, which fact has recently been summarized [28]. One of the issues specific to thyroidectomized patients is the timing of pharmacologic thyroid homeostasis recovery [24]. Recently, a novel means has been proposed, namely, that of predicting the required tailored LT4 dosage after total thyroidectomy using a decision aid tool (DAT) based on pharmacometrics [29]. The authors concluded that the application of a DAT for LT4 dosage would reduce by 1 month the time to attain target TSH as compared with clinician dosage adjustment [29]. However, the process of absorption of LT4 remains partially unknown, several factors potentially hampering the efficacy of LT4 treatment acting both at the gastric and the intestinal level [30]. In fact, although the site of LT4 absorption is the monolayer of enterocytes in the small intestine, there is clinical evidence that the gastric passage also plays a key role [31], therapeutic efficacy being impeded by interfering factors such as dietary interference, concomitant use of specific drugs, gastrointestinal diseases or infections, and/or the biochemical characteristics of LT4 formulations. The latter factors may interfere with absorption both in patients with intact thyroid and in those partially or totally thyroidectomized.

### Dietary interference

Most physicians advise patients to take LT4 in the morning, preferably in a fasting state. However, as mentioned above, physicians and patients should be aware that absorption may be significantly impaired by certain foods and beverages, while, to further ensure optimal absorption, the patient must remain in the fasting state for at least 1 h following LT4 ingestion [32]. While recommendations for morning administration of LT4 remain in force, recently, several studies have demonstrated that bedtime administration can be as effective as that before breakfast (see for rev Ref. 33), a schedule that may, moreover, be of help in improving patients’ compliance with LT4 treatment. The interference exerted by the concomitant ingestion of food and beverages with LT4 sodium may be due to the following three mechanisms: variation of gastric juice pH, absorption of the hormone within the intestine, or interference with intestinal LT4 transporters [9].

The acidity of gastric juice pH in the general population in the fasting state is reported to be between 1 and 2; in the fed state, this value usually rises to 5, subsequently gradually returning to basal value in 3–4 h [34]. An in vitro study clearly showed that dissolution of LT4 decreased substantially for pH over 2.4, reaching a nadir at pH 5 then rising again to a pH over 6 [35]. This finding has been recently confirmed in an in vivo study [31]. The significant reduction of LT4 absorption when the hormone is taken with food has been demonstrated since 1977 [36]. A pharmacokinetic study was recently conducted in 10 patients taking concomitantly LT4 and milk: the co-ingestion led to serum TT4 absorption over 6 h, calculated as AUC; mean peak serum TT4 concentrations were observed to be significantly lower as compared to LT4 ingestion only with water [37]. The precise mechanism causing this reduction of absorption is not as yet known. Besides the food-related changes in gastric pH, it can be hypothesized that the increased need for LT4, is due to the presence in the meal of specific substances that may directly absorb LT4 in the intestinal lumen, subtracting it from the amount available for its absorption [30]. This occurs in the case of a fiber- or soy-enriched diet [38] or in patients experiencing coffee-related increased need for LT4. Namely, an increased need for LT4 has been described following espresso coffee ingestion; in fact, an in vitro study showed that coffee is able to sequester LT4, thus limiting its intestinal absorption [39].

A possible inhibition of intestinal LT4 transporters has been hypothesized with the ingestion of grapefruit juice; the components of grapefruit juice, however, are unlikely to cause the increase in gastric pH and/or the adsorption of LT4 at the intestinal level. On the other hand, the ability of grapefruit juice and other juices to inhibit organic anion-transporting polypeptide-mediated drug uptake inhibition has been demonstrated for fexofenadine, thus decreasing its oral availability [40].

### Concomitant use of other drugs

Polypharmacy is a frequent phenomenon nowadays and may be concomitant in thyroidectomized patients. Calcium supplementation in thyroidectomized patients is one example. The above-described mechanisms are responsible for most of the interference exerted by other drugs ingested concomitantly with LT4 [41]. Proton pump inhibitors represent a model of drugs inhibiting the gastric juice pH, while calcium preparations and sucralfate tend to bind LT4 in the stomach and in the intestine, respectively [30]. An increase in LT4 requirement has been described in thyroidectomized patients treated with TKIs such as imatinib, the authors hypothesizing impaired absorption of LT4 through an as yet unidentified intestinal transporter [42]. Furthermore, several other mechanisms may alter LT4 pharmacological homeostasis, including possibly the following: (a) decreasing TSH secretion (corticosteroids), (b) altering LT4 binding to proteins and/or its transport in serum (estrogens, androgens, and furosemide, (c) modifying peripheral metabolism (carbamazepine, corticosteroids, and beta-adrenergic–antagonist drugs), and (d) interfering with enterohepatic recycling (colestiramine) (see for rev 30 and 41). The pharmacological properties of each drug dictates the appropriate interval to minimize interference with LT4.

### Gastrointestinal disease

Almost all gastrointestinal disorders may affect LT4 pharmacological homeostasis. Clinical evidence of an increased need for LT4 in patients with gastric disease has been reported in chronic atrophic gastritis [31, 43, 44], *Helicobacter pylori* infection [43], and gastroparesis [45], as well as in patients who underwent gastric surgery [46]. Common disorders associated with true malabsorption are celiac sprue and lactose intolerance, together with malabsorptiove bariatric surgery. Case reports have described additional gastrointestinal causes of an increased LT4 dose requirement in patients with pancreatic insufficiency, dysmotility of the distal esophagus, and liver cirrhosis [30]. Notably, the need for a higher LT4 dose was also observed in 12 patients suffering from ulcerative colitis in remission [47]. With regard to lactose intolerance and celiac sprue, it has been reported that starting an appropriate restriction diet might lead to an improvement in LT4 absorption [48–50]. The reversibility of the increased need for LT4 has also been demonstrated following *Helicobacter pylori* infection treatment. Specifically, two studies [43, 51] reported a significant reduction of TSH levels after successful bacterial eradication. However, in one of them [51], 21% of the patients in the study group developed thyrotoxicosis.

### Other disorders

Nephrotic syndrome may increase the need for LT4 due to urinary loss of free and protein-bound thyroid hormones [52].

A diagnosis of DTC during pregnancy may also present a challenge. In this regard, the Endocrine Society advises administering levothyroxine to keep the TSH level suppressed though detectable in high-risk cases should surgery be delayed until after delivery. Moreover, in women previously thyroidectomized for DTC, pregnancy may interfere with LT4 requirement because of the variations in TBG concentration and due to the rearrangement of gastrointestinal physiology (i.e., reduced gastric acid secretion, gastric emptying, and intestinal motility due to high progesterone levels) [53, 54], When these disorders and interference occur in thyroidectomized patients, they may hamper the optimization of LT4 treatment.

## The novel LT4 formulations

Over the last few years, pharmaceutical research has led to the development of LT4 formulations capable of optimizing the efficacy of LT4 treatment in as many patients as possible. One is a liquid preparation in which LT4 is dissolved in glycerol and purified water, ethanol, and glycerol. The other consists of a soft gel preparation in which the active ingredient is dissolved in glycerol and surrounded by a gelatin shell [55]. The advantages of using these preparations have been demonstrated in several categories of patients suffering from various gastrointestinal disorders (gastric atrophy, lactose intolerance, and gastroparesis, to name but a few) [56] and also in the case of interference exerted by food or drugs during concomitant ingestion [57]. One meta-analysis including 141 patients reported that in patients showing suboptimal TSH levels on the tablet, the switch to the same dose of liquid formulation may significantly improve TSH values [58]. Furthermore, another meta-analysis reported that better efficacy may be observed in patients with tablet T4 malabsorption treated both in replacement or suppressive mode [59]. Some studies have examined specifically the usefulness of liquid T4 formulation in patients thyroidectomized and followed up for differentiated thyroid cancer. The first, published in 2015, analyzed the tolerability and efficacy of the liquid as compared to the tablet formulation in 54 treated DTC patients. It is noteworthy that in this sample, the interval between T4 and breakfast ingestion was highly variable. No variations in hormonal pharmacological homeostasis in these patients were observed, although a significant reduction in subjective symptoms was reported [60]. One year later, a study examined the concentration of FT4 following the oral intake of 200 mcg of LT4 in liquid and tablet formulation in 14 patients thyroidectomized for DTC [61]. Specifically, the tablet was taken in the fasting state, while the liquid formulation was ingested with breakfast. Circulating levels of FT4 measured 3–4 h following the tablet (in the fasting state) and the liquid (with breakfast) were similar, indicating the lesser susceptibility of the liquid form to food and beverage interference [61]. In 2017, a study was published examining 102 patients submitted to total thyroidectomy followed by ^131^I remnant ablation for papillary thyroid carcinoma [62]. The patients were subdivided into two groups taking 1.9 µg/kg/day of LT4 in a tablet or a liquid formulation, with the patients taking the liquid preparation during breakfast and those taking the tablet LT4 ingesting it in the fasting state. After 12 and 24 months, the patients taking liquid LT4 exhibited stable TSH (0.28 and 0.30 mU/L) value, while in patients taking LT4 in tablet form, TSH was slightly increased (0.28 and 0.34 mU/L). However, the difference between the latter two TSH values appears to be too small to support a significant difference between these two formulations’ performance in DTC patients. A more recent study [63] compared 52 thyroidectomized DTC patients treated with liquid LT4 formulation and 53 patients treated with tablet LT4. Both groups of patients ingested LT4 30 min before breakfast at a dose of 1.5 mcg/kg/day; no patients showed signs or symptoms attributable to LT4 malabsorption. Thyroid tests were conducted at 6 and 12 weeks and revealed lower TSH values in the group treated with liquid LT4. However, the short delay between LT4 intake and food ingestion might have affected the results.

Thus, the liquid preparation seems to offer some advantage over the tablet form in thyroidectomized patients with concomitant interfering factors, but not in DTC patients without known causes of increased oral LT4 requirement.

## Conclusions

Following thyroid surgery for DTC, the use of an individually tailored LT4 dose must be followed by all clinicians, careful titration of the LT4 dose being even more necessary in this instance than in patients with a nonfunctional thyroid based on the following information:

- The amount of thyroid tissue removed
- The DTC staging, which is mandatory for tailoring the mode and doses of LT4 treatment
- The same dietary, pharmacologic, and pathologic issues applying to patients with or without thyroid
- The need for LT4 following thyroidectomy, which may be increased by 20 to 25%
- The accuracy of LT4 dosage for each patient, appropriate before any treatment switch
- The usefulness of alternative LT4 formulations in thyroidectomized patients with concomitant interfering factors.

Besides the above issues, a highly relevant and as yet unresolved question regards the usefulness and/or timing to start and the dose administered in LT4 in patients undergoing lobectomy and the appropriateness in this instance of combined T4/T3 treatment.
